# Intestinal group 1 innate lymphoid cells drive macrophage-induced inflammation and endocrine defects in obesity and promote insulinemia

**DOI:** 10.1080/19490976.2023.2181928

**Published:** 2023-02-23

**Authors:** Rebeca Liébana-García, Marta Olivares, Carlos Francés-Cuesta, Teresa Rubio, Valerio Rossini, Guillermo Quintas, Yolanda Sanz

**Affiliations:** a Institute of Agrochemistry and Food Technology, Spanish National Research Council (IATA-CSIC), Valencia, Spain; b Health and Biomedicine, Leitat Technological Center, Terrassa, Spain; c Analytical Unit, Health Research Institute La Fe, Valencia, Spain

**Keywords:** Microbiota, obesity, ILC1s, metabolism, high-sucrose high-fat diet, *Akkermansia muciniphila*

## Abstract

Hypercaloric diets overactivate the intestinal immune system and disrupt the microbiome and epithelial cell functions, impairing glucose metabolism. The origins of this inflammatory cascade are poorly characterized. We investigated the involvement of intestinal proinflammatory group 1 innate lymphoid cells (ILC1s) in obesity progression and metabolic disruption. In obese mice, we studied longitudinally the ILC1s response to the diet and ILC1s depletion to address its role in obesity. ILC1s are required for the expansion of pro-inflammatory macrophages and ILC2s. ILC1s depletion induced the ILC3-IL-22 pathway, increasing mucin production, antimicrobial peptides, and neuroendocrine cells. These changes were translated into higher gut hormones and reduced insulinemia and adiposity. ILC1s depletion was also associated with a bloom in *Akkermansia muciniphila* and decreases in *Bilophila* spp. Intestinal-ILC1s are upstream activators of inflammatory signals, connecting immunity with the microbiome, the enteroendocrine system, and the intestinal barrier in the control of glucose metabolism and adiposity.

## Introduction

Obesity is characterized by excessive fat storage that represents the main metabolic risk factor for the development of complications, such as type 2 diabetes (T2D), cardiovascular disease, or cancer. This fact, together with its global relentless advance in the last decades, makes urgent the intensification of the research aim to understand the precise mechanisms by which unbalanced diets trigger obesity. It is well-proven that obesity is associated with chronic inflammation that drives changes in microbiota, glucose metabolism, and insulin resistance.^1^ A change in the microbial configuration *per se* can also modify the equilibrium between different immune cell types and their activation, thereby, increasing the risk of inflammatory pathology driven by a biased-immune response.^2^ In obesity, this loss of intestinal immune homeostasis is an early event that precedes and contributes to systemic inflammation.^3^ While the large excess of energy provided by the diet is ultimately stored as fat in metabolic organs, the intestine is the first compartment affected by obesogenic diets, which can alter the composition and function of intestinal immune cells and the microbiota and their metabolome, in turn, influencing metabolic responses to diet.^4,5^ In fact, this crosstalk between intestinal immune cells and microbiota is essential in shaping immune function.^6^ For example, a fat-enriched diet results in specific changes in the gut microbiota that impair the intestinal immune system and, ultimately, leads to systemic low-grade inflammation.^5^

Innate lymphoid cells (ILCs) are a recently discovered family of immune cells that are mainly localized at epithelial surfaces where they sense intrinsic and extrinsic signals (such as microbes and dietary stimuli) and function to preserve immune homeostasis.^7^ The ILC family compromises natural killer (NK) cells, ILC1s, ILC2s, ILC3s, and lymphoid tissue-inducer (LTi) cells.^8^ Although, immunologists commonly gather them in three groups: ILC1 (comprising ILC1s and NKs), ILC2, and ILC3 (comprising ILC3 and LTi cells), mainly based on the type of immune response they drive.^2^ Emerging evidence links ILCs to obesity through a mechanism that is not fully understood. Obesogenic diets diminish the proportion of intestinal interleukin (IL)-22-producing ILC3s, which might contribute to the gut barrier dysfunction seen in obesity,^9,10^ although uncertainties exist.^11^ In the context of inflammatory bowel diseases, dysregulation of ILC3s has been implicated in their pathogenesis.^12^ Colonic ILC3 mediates a protective response in acute colitis, but sustained intestinal damage leads to a proinflammatory intestinal response mediated by ILC3.^13^ A protective role for ILC2s in adipose tissue has been reported in mice and humans,^14^ and group 1 ILCs have been described to regulate and polarize macrophages to drive inflammation, which promotes insulin resistance.^15–17^ Also, another study reported that intestinal ILC2s, but not ILC2s from adipose tissue, are involved in obesity development in mice fed hypercaloric diets.^18^ This unequivocally points to the tissue-specific functions played by this predominantly tissue-resident family of immune cells. So far, the implications of adipose ILC1s in obesity and metabolic complications have been widely evidenced, but the potential function of the intestinal-group 1 ILCs awaits further investigations.

We hypothesized that gut-resident ILC1s increase in abundance as a consequence of hypercaloric diets, and initiate a cascade of pathogenic events that stimulate inflammation and trigger intestinal barrier and enteroendocrine cell dysfunction. Given the links between the microbiota and immunity, we further postulated that ILC1-triggered inflammation suppresses host-microbiota symbiosis, which would account for the systemic dysregulation of glucose homeostasis. Here, we present intestinal ILC1s implication in the cascade of events promoting inflammation in obesity and its links to intestinal and endocrine homeostasis. As well, we have evidence of the impact of the depletion of intestinal ILC1s on the microbiota and the metabolic profile.

## Results and discussion

### Obesogenic diet boosted ILC1s in the gut closely linked to the expansion of the adipose tissue

**Figure 1.**
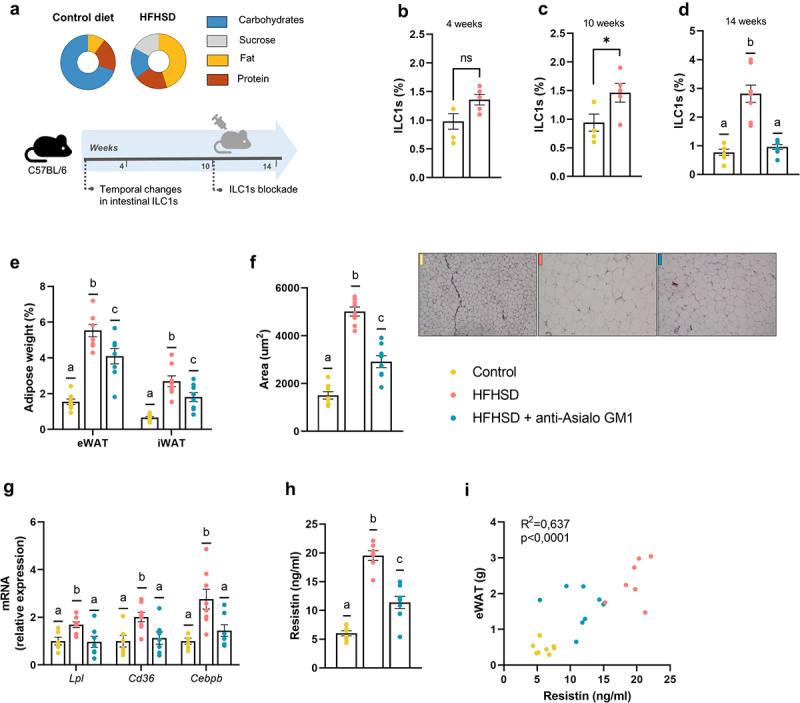
Obesogenic diet boosted ILC1s in the gut and adipose tissue expansion countered by ILC1s depletion (a) Schematic representation of the experimental design. (b–d) ILC1s (percentage of LIN^−^ cells of total intestinal epithelial cells) in the small intestine at 4 weeks, 10 weeks, and 14 weeks, respectively (n = 5–8). (e) Weight of epididymal and inguinal white adipose tissue (eWAT and iWAT, respectively) (n = 8). (f) adipocyte area (n = 8) and representative images of the histological staining of visceral adipose tissue (8×). (g) Expression of markers of fatty acid uptake and adipogenesis, *Lpl, Cd36* and *Cebpb* (n = 7–8). (h,i) Plasma resistin levels and correlation between resistin levels and eWAT content (n = 7–8). Bars represent the mean ± SEM and biological replicates are shown as individual dots. For normally distributed data statistical analyses were performed by t-test or ANOVA, as appropriate, followed by *post hoc* Tukey’s multiple comparison test. Welch’s correction was applied when variances were not equally distributed. Non-normally distributed data were analyzed with the Mann-Whitney or Kruskal-Wallis test (as appropriate) followed by Dunn’s multiple comparisons test. Correlations were calculated with Pearson’s test. “*” p < .05. Data with different superscript letters are significantly different at p < .05.

We first investigated the effect of a high-fat, high sugar-diet (HFHSD 45% of energy from lard, and 17% from sucrose) on intestinal ILC1s, expecting that, similar to adipose tissue ILC1s,^19^ gut ILC1s will dynamically respond to dietary changes. Additionally, in order to test our hypothesis, we used a neutralizing antibody to deplete ILC1s in male C57BL/6 mice fed an HFHSD (Figure 1a). Specifically, we chose the anti-Asialo GM1 (AGM1) that targets the asialo-GM1, a cell-surface glycolipid present in ILC1 group (referred to as ILC1s hereinafter). This is a well-described strategy for blocking ILC1s function, but not exempt from limitations, such as the possible off-target interactions with other cells and the lack of tissue specificity.

We found that fat mass was greater in mice on HFHSD than on a control diet after 4 weeks, whereas no significant changes were observed for body weight or small intestine ILC1s abundance (Figure 1b, Supplemental Figure S1a, b). By contrast, ten weeks of HFHSD increased gut ILC1s abundance, body weight, and fat mass (Figure 1c, Supplemental Figure S1c, d), which is consistent with a previous study reporting an increase in intestinal ILC1s abundance in mice fed an obesogenic diet for 12 weeks.^20^ Similar to the observations with obesogenic diets, inflammatory bowel diseases such as Crohn’s disease and ulcerative colitis course with elevated intestinal ILC1s abundance.^21,22^ Interestingly, no increase in ILC1s was observed in non-inflamed tissue in patients without inflammatory bowel disease, unequivocally linking ILC1s with an inflammatory milieu.^22^ To study the role of ILC1s in obesity, we treated mice with an AGM1 antibody, which depletes ILC1s,^15,19^ from weeks 10 to 14 of HFHSD. No changes in food intake were observed in mice treated or not with AGM1 during this period when compared with mice on a control diet (Supplemental Figure 1e). As expected, the abundance of ILC1s was significantly lower in AGM1-treated mice than in non-treated mice (Figure 1d). Additionally, adiposity in two fat deports (epididymal [eWAT] and inguinal white adipose tissue) was significantly lower in AGM1-treated mice than in non-treated mice (Figure 1e), which was accompanied by a tendency for reduced body weight gain (Supplemental Figure 1f). Likewise, adipocyte size was significantly smaller in AGM1-treated mice (figure 1f), consistent with a downregulation in the expression of genes involved in fatty acid uptake (*Lpl* and *Cd36*) and adipogenesis (*Cebps*), which were all increased by HFHSD (Figure 1g). In agreement with these data, previous studies point to ILC1s as major promoters of adipose tissue hypertrophy and inflammation.^17,23^ Specifically, ILC1s have been shown to promote CD11c^+^ macrophage activation resulting in adipose fibrogenesis, an indicator of advanced obesity;^17^ other authors, however, failed to find a link between ILC1s and adiposity in animal models.^15,16^ We also found that ILC1s depletion decreased significantly the plasma levels of resistin and tended to reduce those of leptin, both well-recognized adipokine markers of adiposity^24,25^ (Figure 1h). Resistin was positively correlated to eWAT mass (Figure 1i). Overall, our results indicate that gut-resident ILC1s increase in response to HFHSD and likely contribute to several features of obesity.

### ILC1s depletion reduced intestinal proinflammatory response and protects from the gut homeostasis breakdown

**Figure 2.**
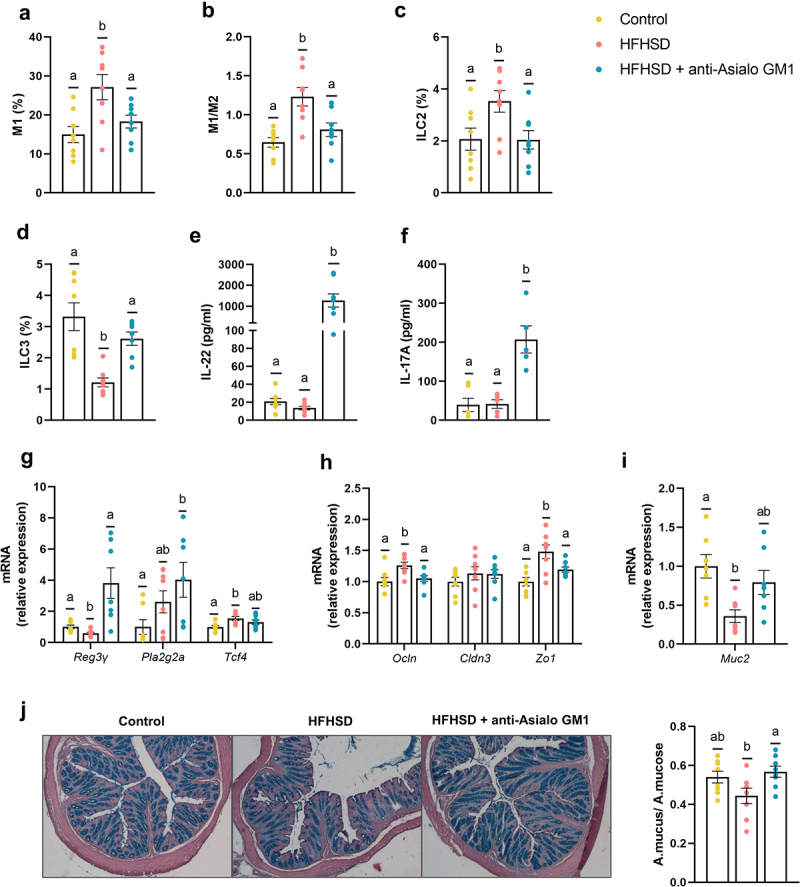
Depletion of intestinal ILC1s improved gut homeostasis. (a) Pro-inflammatory macrophages (M1) (b) ratio of M1/M2 macrophages (n = 8) in the small intestine. (c,d) ILC2s and ILC3s (percentage of LIN^−^ cells of the total lamina propria cells) in the small intestine (n = 7–8). (e,f) Plasma levels of IL-22 and IL-17A (n = 5–8). (g) Ileal gene expression of antimicrobial peptides (*Reg3g*, and *Pla2g2a*), the Paneth differentiation marker *Tcf4* and, (h) tight junction markers (*Ocln, Cldn3* and *Zo1*). (i) Colonic expression of *Muc2*, and (j) representative images of the histological staining of the colonic tissue with alcian-blue and quantification of mucus production as the area of mucus relative to the area of the total mucosa. Bars represent the mean ± SEM and biological replicates are shown as individual dots. Statistical analyses were performed by ANOVA followed by *post hoc* Tukey’s multiple comparison test for normally distributed data. Welch’s correction was applied when variances were not equally distributed. Non-normally distributed data were analyzed with the Kruskal-Wallis test followed by Dunn’s multiple comparisons test. Data with different superscript letters are significantly different at p < .05.

Given these findings, we next investigated the dialog between ILC1s and macrophages in the gut. This immune-cell interaction has been studied mainly in obese adipose tissue.^16,19^ As anticipated, HFHSD feeding for 14 weeks created a pro-inflammatory milieu that boosted the expansion of type 1 (M1) macrophages and the M1/M2 ratio together with ILCs in the gut, as revealed by flow cytometry analysis (Figure 2a,b). Additionally, ILC1s depletion blunted the expansion of M1 macrophages and reduced the M1/M2 ratio in the gut (Figure 2a,b). No diet or antibody-induced changes were observed for M2 macrophages (Supplemental Figure S2a). These results indicate that activation of ILC1s is obligatory for the subsequent expansion of pro-inflammatory M1 macrophages in the gut.^19,23^ The changes in the inflammatory tone due to ILC1s depletion secondary impacted other ILC subsets. Recent studies have reported an increase in the proportion of ILC2s in high-fat diet-induced obesity.^18^ We found that ILC1s depletion suppressed the HFHSD-induced increase in ILC2 abundance, with levels equal to those in the control diet group (Figure 2c). Contrastingly, we observed a reduction in ILC3s in HFHSD-fed mice and an increase in the obese group treated with AGM1 (Figure 2d). ILC3s are involved in tissue-protective responses to damage,^8^ and an increase in ILC1s in inflamed tissue has been linked to a reduction in ILC3s as a consequence of a shift from the latter to the former.^21^ We noted the same relationship between ILC1s and ILC3s in the obesity model; however, our experimental approach does not allow us to assess this phenomenon directly. To support these findings, we measured the plasma levels of IL-22 and IL-17A, two effector cytokines of ILC3s. At 10 weeks of HFHSD, IL-22 levels were significantly lower than those in the control diet group (Supplemental Figure S2b). No differences were observed at 14 weeks between the control diet and the HFHSD-fed groups; however, ILC1s depletion led to an increase in the plasma levels of both IL-22 and IL-17A (Figure 2e,f). Furthermore, IL-22 and IL-17 showed increased expression in the ileum, reflecting how intestinal ILC1s depletion affects those cytokines also in the intestine (Supplemental Figure S2c). The ILC3/IL-22 pathway bolsters gut barrier function by inducing the production of antimicrobial peptides (AMPs) and mucin.^10^ Accordingly, ILC1s depletion led to a significant increase in the ileal gene expression of the AMP regenerating islet-derived 3-gamma (*Reg3g*) in HFHSD-fed mice, which was accompanied by a trend for an increase in the expression of phospholipase A2g2 (*Pla2g2a*). This response occurred without changes to the expression of *Tcf4*, a transcription factor involved in the differentiation of AMP-producing Paneth cells (Figure 2g). In the colon, no differences were found (Supplemental Figure S2d). The lack of an immune response mediated by ILC1s also influenced the expression of two of the three tight junction protein genes analyzed (*Zo1* and *Ocln*) in the ileum (Figure 2h), whereas, in the colon, only *Ocln* showed a trend to be increased in mice treated with the antibody AGM1 compared to untreated obese mice Supplemental Figure S2d). Moreover, AGM1 treated mice presented an increase in mucin (*Muc2*) expression and mucus production (Figure 2i,j), which could be secondary to the increase in ILC3 abundance.^10^ Collectively, these findings indicate that the pro-inflammatory microenvironment driven by ILC1s impairs different mechanisms involved in strengthening the gut barrier. Interestingly, some of the effects of ILC1s depletion have been, similarly, attributed to anti-obesogenic treatments including prebiotics or vegetable-rich extracts.^26^,^27^

### ILC1s depletion alleviated gut hormones dysregulation which could improve glucose metabolism

**Figure 3.**
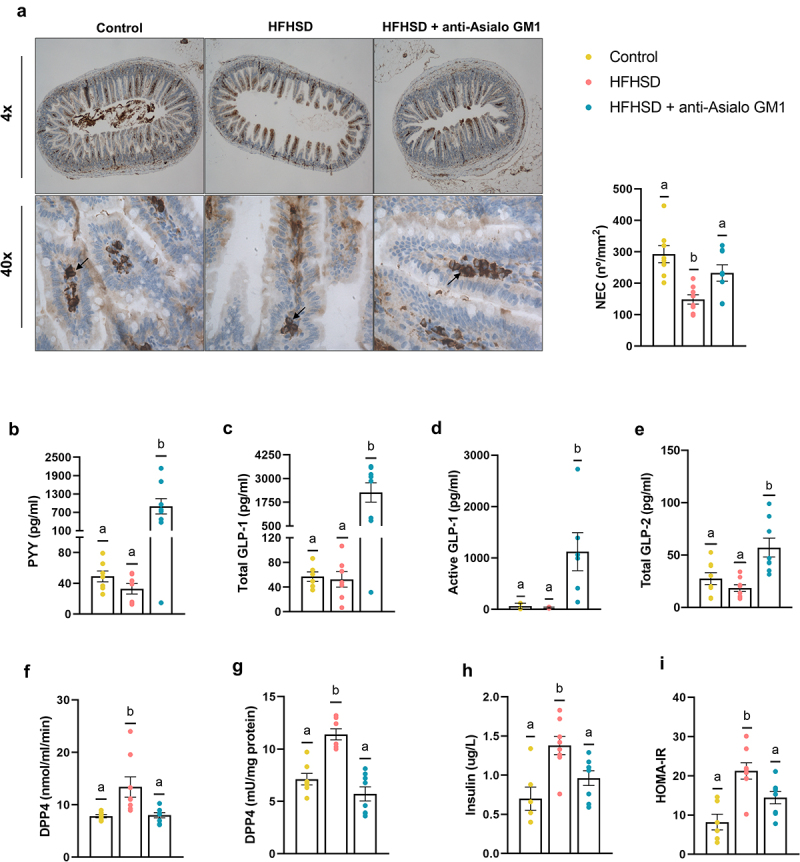
Depletion of ILC1s influenced enteroendocrine control. (a) Immunolabeling of ileal sections and the number of immune-positive neuroendocrine cells per mucosa area (n = 8). (b) Plasma levels of the hormones PYY (n = 7–8), (c) total GLP-1 (d) and active GLP-1 (n = 6–8), and (e) total GLP-2, (f) DPP4 activity in plasma and in (g) visceral adipose tissue (n = 6–8). (h) Insulinemia (n = 6–8) and (i) HOMA-IR index (n = 6–8). Bars represent the mean ± SEM and biological replicates are shown as individual dots. Statistical analyses were performed by ANOVA followed by *post hoc* Tukey’s multiple comparison test for normally distributed data. Welch’s correction was applied when variances were not equally distributed. Non-normally distributed data were analyzed with the Kruskal-Wallis test followed by Dunn’s multiple comparisons test. Active GLP-1 was analyzed by Chi-square contingency tests. Data with different superscript letters or the symbol “*” show significant differences at p < .05.

We further investigated the consequences of ILC1s depletion on the enteroendocrine system to explore possible links to obesity. Guided by previous evidence, we found a relationship between endocrine hormones and intraepithelial lymphocytes. Immunohistochemical analysis of the ileum revealed that neuroendocrine cells (NECs) were less abundant in the HFHSD-fed group than in the control diet group, whereas the opposite was seen in AGM1-treated mice on HFHSD (Figure 3a). The expression of neuronal differential 1 (*NeuroD1*), but not neurogenin 3 (*Ngn3*) (factors related to NEC development and differentiation) negatively correlated with NEC abundance, likely as a compensatory response (Supplemental Figure S3a). We then questioned whether changes in NECs led to differences in circulating gut hormones involved in glycemic regulation. Although we found no differences in response to the diet, ILC1s depletion caused a sharp rise in peptide YY (PYY) and in glucagon-like peptide 1 (GLP-1) (total and active) and total GLP-2 (Figure 3b–e). In addition to the canonical role of GLP-1 in glucose homeostasis, its role in buttressing the gut barrier has been recently established,^28^ similar to the role attributed to GLP-2.^29^ Depletion of ILC1s also tended to restore the levels of the gastric inhibitory polypeptide (GIP) to those in the control diet group (Supplemental Figure S3b). Incretins are inactivated by dipeptidyl-peptidase 4 (DPP4), a ubiquitous protease closely linked to obesity.^30^ In line with previous observations, we found an increase in plasma DPP4 activity in HFHSD-fed mice, but this effect was not observed in AGM1-treated mice (figure 3f) and was not a direct effect of the AGM1 antibody (Supplemental Figure S3c). We next investigated the possible origin of the higher circulatory DPP4 activity. Adipose tissue is a major source of DPP4, where it modulates key metabolic features of obesity including inflammatory tone, glucose homeostasis, and adiposity.^31^ The obesogenic diet increased DPP4 activity in eWAT, and this was prevented by AGM1 treatment (Figure 3g). No differences were found in liver, another important source of DPP4 activity (Supplemental Figure S3e). Then, the observed increase in active GLP-1 can be explained by the greater abundance of NECs in the intestine and the diminished adipose and plasma DPP4 activity. In agreement with previous studies,^15,16,23^ ILC1s depletion rescued the hyperinsulinemia caused by the HFHSD and the homeostatic model assessment for insulin resistance (HOMA-IR) (Figure 3h, i). Overall, these studies allow us to hypothesize that the increased intestinal hormone concentrations secondary to ILC1s depletion in the intestine explain the observed improvement in gut homeostasis and insulinemia.

### ILC1s depletion imprinted microbiota and metabolic changes toward a healthier intestinal microenvironment

**Figure 4.**
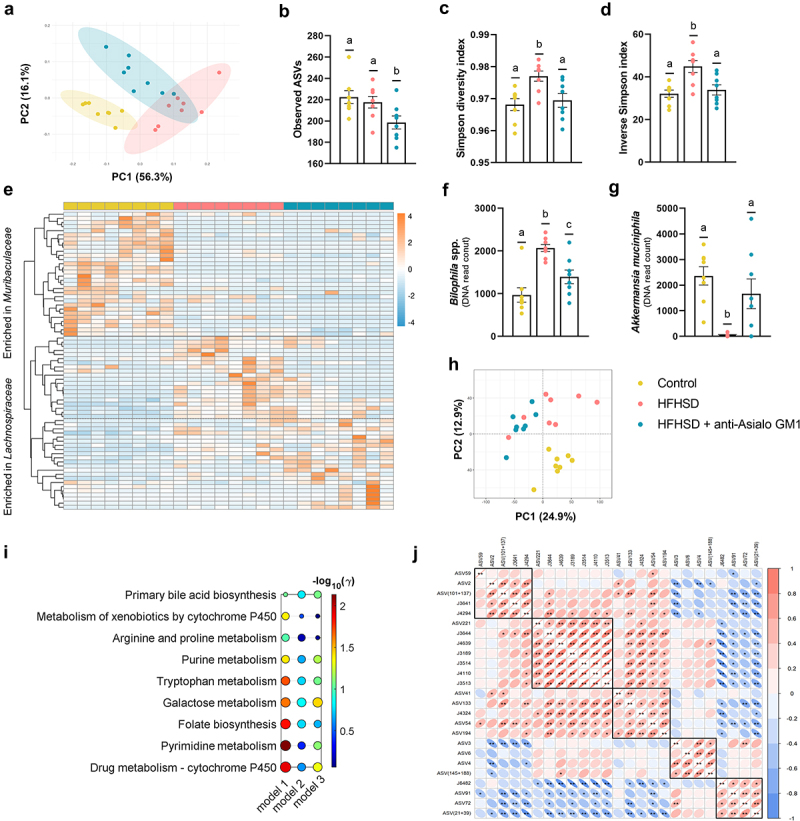
Depletion of intestinal ILC1s shaped the gut microbiome and the metabolic signature. (a) Beta-diversity based on weighted UniFrac distances. (b) Observed ASVs. (c, d) Simpson’s diversity and evenness indexes. (e) Heat-map representing the clustering of the two more abundant families (*Muribaculaceae* and *Lachnospiraceae*). (f) *Bilophila* spp. and (g) *Akkermansia muciniphila* abundance. (h) Principal component analysis based on metabolomic data. (i) Scheme of the altered pathways between HFHSD-fed animals and HFHSD-fed mice depleted for ILC1s (model 1: HFHSD vs HFHSD anti-Asialo GM1; model 2: control diet vs HFHSD anti-Asialo GM1; model 3: control diet vs HFHSD), and (j) correlations between the ASVs and the metabolites significantly different between HFHSD vs HFHSD anti-Asialo GM1. ASV59: *Lachnospiraceae* NK4A136 group; ASV2: *Bilophila*; ASV (101 + 137): *Colidextribacter*; J3641: uridine; J4294: glucosiduronic acid; ASV221: *Lachnospiraceae* GCA-900066575; J3644: Fatty acid; J4639: D-maltose; J3189: suberic acid; J3514: sedanonic acid; J4110: quesitol; J3513: traumatic acid; ASV41: *Oscillospiraceae*; ASV133: *Oscillospiraceae* UCG-003; J4324: Fatty acyls; ASV54: *Lachnospiraceae* NK4A136 group bacterium; ASV194: *Lachnospiraceae* A2; ASV3: *Akkermansia muciniphila*; ASV6: *Rikenellaceae* RC9 gut group; ASV4: *Lachnospiraceae* NK4A136 group bacterium; ASV (145 + 188): *Ruminococcaceae*; J6482: rutin; ASV91: *Coriobacteriaceae* UCG-002; ASV72: *Parasutterella*; ASV (21 + 39): *Bacteroides*. In all cases, n = 8. Bars represent the mean ± SEM and biological replicates are shown as individual dots. Pathway analysis was carried out using the mummichog algorithm with a p-value cutoff of 0.05 and the human KEGG pathway database.

Finally, we investigated whether the gut microbiota composition and function are affected by ILC1s depletion. According to principal coordinates analysis (PCoA) of the weighted UniFrac distances, 14 weeks of HFHSD caused an evident structural change in the microbial community, explained by the PC1 (56.3% of the variability). The PC2 (16.1%) can be attributed to ILC1s depletion (Figure 4a). The AGM1 antibody also reduced the observed amplicon sequence variants (ASVs) as compared with mice fed a control diet or HFHSD (Figure 4b), and normalized the Simpson’s diversity and evenness indices (Figure 4c,d). Overall, we observed that the families that best discriminated between the three experimental groups were *Muribaculaceae* and *Lachnospiraceae* (Figure 4e). Both groups of mice fed an HFHSD present reductions in ASV of the Muribaculaceae family. In the Lachnospiraceae family, two clusters can be distinguished. The ASVs of the first cluster were increased by the obesogenic diet and restored by the ILC1s depletion, while in the other cluster we observed an increase in several ASV associated with the ILC1s. Among the ASVs (identified at genus or species levels) that were significantly modified by ILC1s depletion *versus* HFHSD only, two ASVs were prominent: *Bilophila* spp. and *Akkermansia muciniphila. Bilophila* spp. is known to synergize with an obese diet, promoting intestinal inflammation and gut barrier dysfunction.^32^ Increases in *Bilophila* spp. were counteracted by ILC1s depletion (figure 4f). By contrast, *A. muciniphila* abundance was blunted by the HFHSD and recovered after ILC1s depletion (Figure 4g). *A. muciniphila* plays a key role in metabolic health and is generally associated with a lean phenotype; indeed, its administration neutralizes several obesity hallmarks.^33^ Other genera affected by the antibody treatment are shown in Supplemental Figure S4a–h. We also conducted a non-targeted metabolomic analysis of the cecal content. In total, 6984 different metabolic features were detected, after peak detection and clean-up, among which the mean value of 2387 features was significantly altered (Supplemental Table S1). An exploratory PCA revealed that most of the changes were caused by ILC1s depletion (24.9% of explained variance), although, as expected, the HFHSD also triggered a shift in the metabolic profile (12.9%) (Figure 4h). From the total detected features, only 263 (~4%) were adequately annotated, with several notable examples. Imidazole propionate is a novel microbial-derived metabolite associated with T2D and is elevated in patients with gut inflammation.^34^ We found that imidazole propionate was significantly decreased by ILC1s blockade as compared with the control diet group but not with the HFHSD group (Supplemental Figure S4i). Moreover, a significant increase in the dietary flavonoid rutin was evident in the AGM1-treated group (Supplemental Figure S4j). It has been shown that rutin supplementation in obese mice increases serum GLP-1 levels and dose-dependently inhibits DPP4 activity.^35^ A deeper metabolic network analysis using the *mummichog* algorithm revealed 9 altered pathways (containing at least 3 tentatively assigned metabolites and more than 3 significantly altered) between HFHSD-fed animals treated or not with AGM1 (model 1) (Figure 4i). In total, 7 pathways were significantly modified (γ-value < 0.05), of which 2 also appeared significantly altered in model 3 (control vs HFHSD) (Figure 4i, Supplemental Table S2). No pathways were significantly altered in model 2 (control vs HFHSD+AGM1) (Figure 4i, Supplemental Table S2). The greatest metabolic change in model 1 was found in the pathway of pyrimidine metabolism. Alterations in pyrimidine metabolism have been associated with lipid accumulation and gestational diabetes.^36,37^ Among the 34 metabolites tentatively assigned to the pyrimidine pathway, uridine was one of those identified (Supplemental Table S2). The role of uridine in obesity remains controversial. Uridine administration can ameliorate diet-induced obesity,^38^ but high uridine plasma levels are related to obesity and T2D.^39^ We found that uridine levels were reduced by ILC1s depletion (Supplemental Figure S4k). Additionally, the analysis revealed that uridine was positively correlated with *Bilophila* spp., *Colidextribacter* spp., and *Lachnospiraceae* NK4A136 group bacterium (a member of the *Lachnospiraceae* family), which we associated with the obese phenotype, and negatively correlated with *A. muciniphila* (Figure 4j). Overall, there is an improvement in the microbial ecosystem secondary to the depletion of ILC1s and the pro-inflammatory intestinal milieu in obesity. Given that non-targeted metabolic analysis lacks an initial hypothesis, some of our conclusions remain speculative; however, our approach allowed us to characterize metabolite changes associated with the microbiota and ILC1s depletion that warrant further investigation.

## Conclusions

We establish, for the first time to our knowledge, the contribution of intestinal ILC1s to the activation of macrophage-induced inflammation, with downstream consequences for endocrine peptides responsible for metabolic dysregulation in obesity. We cannot, however, exclude the possibility that AGM1 can reach other tissues (like the adipose and the liver^19,40^) and some of our data (such as the lower adiposity or improved insulin resistance index) might also be an effect of ILC1s depletion in other locations. Nonetheless, we confirmed that the depletion of intestinal ILC1s coincided with the restoration of intestinal immune and endocrine markers compromised by the obesogenic diet. Moreover, ILC1s depletion prevented changes to the integrity of the mucus barrier through activation of the ILC3/IL-22 pathway and reset the microbiota and metabolome profiles to a healthier symbiotic state. It remains to be established whether intestinal ILC1s might be a primary target to alleviate unhealthy diets effects and improve or prevent the metabolic complications in obesity.

## Methods

### Mice and treatments

Experiments were performed using a total of 44 male C57BL/6 J mice (7 weeks of age; Charles River Laboratories, Écully, France). Mice were housed in groups of 4/5 per individually-ventilated cage under a 12-h light/dark cycle, in temperature-controlled conditions (23 ± 2°C). Mice were acclimatized for 10 days and had *ad libitum* access to water and food. Mice were randomized based on body weight to minimize baseline differences and were exposed for 4, 10 or 14 weeks to: (i) control diet (D12450K Ssniff Spezialdiäten GmbH, Soest, Germany; 10% of energy from fat and no sucrose), or (ii) HFHSD (D12451 Ssniff; 45% of energy from lard, and 17% from sucrose). From week 10 to 14, a subgroup of mice on the HFHSD (n = 8) was treated by intraperitoneal injection with 50 µl of the AGM1 antibody (Biolegend, San Diego, CA) following manufacturer’s instruction and twice weekly according to Lee et al.^15^ Control groups received PBS instead. Body weight evolution and food intake were monitored twice weekly.

After 4 and 10 weeks of treatment, 5 animals of each experimental group were anesthetized with isoflurane and sacrificed by cervical dislocation under fasted conditions.

The remaining 24 animals were sacrificed at week 14. To minimize potential confounders, the sacrifice order was designed so mice of different experimental groups were interspersed. Blood was collected in EDTA-containing tubes with or without a DPP4 inhibitor (Millipore, Burlington, MA). The plasma was recollected after centrifugation (12,000 × g, 3 min) and stored at −80°C. Samples of WAT, ileum and colon were fixed in 4% paraformaldehyde for histological analyses. Liver, WAT (subcutaneous [inguinal] and visceral [epididymal]), ileum, colon, and cecal content were snap-frozen in liquid nitrogen and stored at −80°C until use.

The experiment was approved and performed following European Union 2010/63/UE and Spanish RD53/201 guidelines, was approved by the ethics committee of the University of Valencia (Animal Production Section, SCSIE, University of Valencia), and was authorized by the competent authority (Generalitat Valenciana). The procedure was entitled “*Influence of innate lymphoid cells in obesity development*” and assigned the code 2020/VSC/PEA/0114.

### Biochemical analysis

In the plasma containing the DPP4 inhibitor, the levels of PYY, active and total GLP-1, GIP, insulin, resistin and leptin were measured using the Luminex™ Mouse Metabolic Hormone Expanded kit (Merck Chemicals and Life Science, Madrid, Spain). Total GLP-2 was quantified using a commercial kit (Abyntek Biopharma, Bizcaia, Spain). We also quantified the levels of glucose (Química Analítica Aplicada SA, Spain). The homeostatic model assessment for insulin resistance (HOMA-IR) index was calculated as fasting plasma insulin (mU/L) × fasting plasma glucose (mmol/L)/22.5.

### DPP4 activity

The DPP4 activity was measured in different samples (liver, adipose tissue and, plasma). The inhibitory DPP4 activity of the AGM1 antibody was also investigated using a pool of plasma as a source of DPP4 activity. The DPP-4 was measured as previously described^41^ and detailed in Supplementary Material.

### Immune parameters

Plasma levels of IL-22 and IL-17A were measured using the Luminex™ Mouse Th17 Bead Panel kit (Merck Chemicals and Life Science, Madrid, Spain).

### Isolation of intestinal immune cells and flow cytometry analysis

Cell isolation was performed as described.^42^ Briefly, the small intestine was washed with cold PBS, longitudinally opened, and cut into small pieces. The epithelium and the lamina propria were isolated, digested, and stained with the corresponding antibodies as described in Supplementary Material. Data acquisition and analysis were performed using a BD LSRFortessa flow cytometer operated with FACS Diva software v.7.0 (BD Biosciences). Data were analyzed using FCS express version 5.

### Gene expression analyses

Total RNA was isolated from different sections of the intestine (ileum and colon) using a commercial kit (Nucleo Spin RNA, Nordrhein-Westfalen, Germany) and from the WAT using the TRIsure Reagent (Bioline, London, UK). Complementary DNA was prepared by the reverse transcription of 1 µg of total RNA using a High-Capacity cDNA Reverse Transcription kit (Applied Biosystems, Foster City, CA). Reactions contained the LightCycler 480 SYBR Green I Master mix (Roche, Boulogne-Billancourt, France) and 300 nM of gene-specific primer pairs. RT-qPCR was performed with the LightCycler® 480 Instrument (Roche).^43^ Data were analyzed using the 2^−ΔΔCT^ method. Targeted genes were normalized against ribosomal protein L19 (*Rpl19*) as the housekeeping gene; primer sequences are detailed in Supplemental Table S3.

### Histological and immunohistochemical analysis

Adipocyte size was quantified in visceral WAT after staining with hematoxylin/eosin. Bright-field digital images were taken using an Eclipse 90I (E90I) Nikon microscope (Nikon Corp., Tokyo, Japan) and analyzed as previously described.^43^ NECs in the ileum were detected in paraffin sections (5 μm) immunostained with an antibody to synaptophysin, using the Autostainer link 48 (DAKO, Glostrup, Denmark). The results were expressed as the average NEC number per mucosal area (number/mm^2^). Mucin production in the colon was calculated after staining with Alcian blue, using eosin as a contrast dye. Mucin production was expressed as the total area of mucin-producer cells relative to the whole mucosa area. Histological and immunohistochemical analyses involved a specialist-blinded researcher. All detailed protocols can be found in Supplementary Material.

### Metabolomic analysis

The cecal content was resuspended in methanol (750 mg/ml) and centrifuged at full speed (15,000 x g, 10 min), and filtered (0.22 µm) . Metabolites were studied by a non-targeted approach using a UPLC chromatograph (Agilent Technologies, Santa Clara, CA). Full scan MS data from 100 to 1700 *m/z* was collected on an iFunnel quadrupole time-of-flight (TOF) Agilent 6550 spectrometer (Agilent Technologies). Detailed information and experimental settings for every step of the analysis are in **Supplementary Material.**

### DNA extraction and sequencing

DNA from the cecal content of the 14-week-old group (n = 24) was extracted using the QIAmp® Fast DNA Stool Mini Kit (Qiagen, Hilden, Germany). Library preparation was performed using Nextera XT v2 Index (Illumina, San Diego, CA) targeting the V3-V4 region of the 16S rRNA gene, and sequenced on an Illumina® MiSeq platform (2 × 300 bp paired-end reads).

### Intestinal bacterial diversity and taxonomic analysis

Raw reads were filtered for quality assurance and clean pairs of reads were merged into contig sequences. An ASV table was constructed, and chimeric sequences were removed. Taxonomy was assigned by checking sequences with the SILVA v.138 database. All steps were performed using DADA2 v.1.24 package in R v.4.2. Taxa with a prevalence below 5% were removed from subsequent analyses. The alpha diversity within samples was computed through the estimation of the richness (observed potential species), Simpson, and the inverse of Simpson indices using Phyloseq v.1.40. A maximum-likelihood phylogenetic tree of ASVs was constructed using the general time reversible substitution model with four gamma categories using Phangorn v.2.8.1. The phylogenetic tree was used to calculate the weighted UniFrac distance for computing differences between microbial communities using Phyloseq. Microbiome differential abundance analysis was performed using DESeq2 v.1.36 R package. The DESeq2 implemented function was used to normalize the data and hypothesis testing was performed using the Wald test. The resulting p-values were corrected using the BH-FDR procedure.

### Statistical analysis

G*Power 3.1.9.2 was used to calculate the sample size allowing the primary outcomes (adiposity and glucose metabolism). Data from biochemical, histological, and gene expression studies were analyzed using GraphPad software (v.9 San Diego, CA). The Shapiro-Wilk test was employed to assess data normality. The differences for normally distributed data were determined using one-way analysis of variance (ANOVA) or two-way ANOVA followed by *post hoc* Tukey’s multiple comparison tests or t-test (as suitable). Welch’s correction was applied when variances were not equally distributed. Non-normally distributed data were analyzed using the Mann-Whitney or Kruskal-Wallis test (as suitable) followed by Dunn’s multiple comparisons test. A χ2 test was used for categorical data (active GLP-1). Correlations between resistin and eWAT were calculated with the Pearson test. The Grubbs test was used for outlier detection. For all analyses, results were expressed as mean ± SEM and considered statistically significant at p < .05. Data analysis was not blinded.

## Supplementary Material

Supplemental Material

## Data Availability

The sequences corresponding to the analyses of the gut microbiota composition are uploaded in the ENA (project number PRJEB53615; https://www.ebi.ac.uk/ena/browser/view/PRJEB53615). The non-targeted metabolic data used in this study is available in the Zenodo repository (https://zenodo.org/record/7104987#.Y_OwfR_MJdg, doi:10.5281/zenodo.7104987).

## References

[1] Roselli M, Canali R, Finamore A, Ghiselli A, Devirgilis C. Immune system, gut microbiota and diet: an interesting and emerging trialogue. IntechOpen. 2022. doi:10.5772/intechopen.104121.

[2] Eberl G. Immunity by equilibrium. Nat Rev Immunol. 2016;16:524–14. doi:10.1038/nri.2016.75. 27396446

[3] Winer DA, Luck H, Tsai S, Winer S. The intestinal immune system in obesity and insulin resistance. Cell Metab. 2016;23:413–426. doi:10.1016/j.cmet.2016.01.003. 26853748

[4] Liébana-García R, Olivares M, Bullich-Vilarrubias C, López-Almela I, Romaní-Pérez M, Sanz Y. The gut microbiota as a versatile immunomodulator in obesity and associated metabolic disorders. Best Pract Res Clin Endocrinol Metab. 2021;35:101542. doi:10.1016/j.beem.2021.101542. 33980476

[5] Garidou L, Pomié C, Klopp P, Waget A, Charpentier J, Aloulou M, Giry A, Serino M, Stenman L, Lahtinen S, *et al*. The gut microbiota regulates intestinal CD4 T cells expressing RORγt and controls metabolic disease. Cell Metab. 2015;22:100–112. doi:10.1016/j.cmet.2015.06.001. 26154056

[6] Khan S, Luck H, Winer S, Winer DA. Emerging concepts in intestinal immune control of obesity-related metabolic disease. Nat Commun. 2021;12:2598. doi:10.1038/s41467-021-22727-7. 33972511 PMC8110751

[7] Gury-BenAri M, Thaiss CA, Serafini N, Winter DR, Giladi A, Lara-Astiaso D, Levy M, Salame TM, Weiner A, David E, *et al*. The spectrum and regulatory landscape of intestinal innate lymphoid cells are shaped by the microbiome. Cell. 2016;166:1231-1246. 10.1016/j.cell.2016.07.043 27545347

[8] Vivier E, Artis D, Colonna M, Diefenbach A, Di Santo JP, Eberl G, Koyasu S, Locksley RM, McKenzie ANJ, Mebius RE, *et al*. Innate lymphoid cells: 10 years on. Cell. 2018;174:1054–1066. doi:10.1016/j.cell.2018.07.017. 30142344

[9] Wang X, Ota N, Manzanillo P, Kates L, Zavala-Solorio J, Eidenschenk C, Zhang J, Lesch J, Lee WP, Ross J, *et al*. Interleukin-22 alleviates metabolic disorders and restores mucosal immunity in diabetes. Nature. 2014;514:237–241. doi:10.1038/nature13564. 25119041

[10] Zou J, Chassaing B, Singh V, Pellizzon M, Rici M, Fythe MD, Kumar MV, Gewirtz AT. Fiber-mediated nourishment of gut microbiota protects against diet-induced obesity by restoring IL-22-mediated colonic health. Cell Host Microbe. 2018;23:41–53. doi:10.1016/j.chom.2017.11.003. 29276170 PMC6005180

[11] Mao K, Baptista AP, Tamoutounour S, Zhuang L, Bouladoux N, Martins AJ, Huang Y, Gerner MY, Belkaid Y, Germain RN, *et al*. Innate and adaptive lymphocytes sequentially shape the gut microbiota and lipid metabolism. Nature. 2018;554:255–259. doi:10.1038/nature25437. 29364878

[12] Zeng B, Shi S, Ashworth G, Dong C, Liu J, Xing F. ILC3 function as a double-edged sword in inflammatory bowel diseases. Cell Death Dis. 2019;10:315. doi:10.1038/s41419-019-1540-2. 30962426 PMC6453898

[13] Castellanos JG, Woo V, Viladomiu M, Putzel G, Lima S, Diehl GE, Marderstein AR, Gandara J, Perez AR, Withers DR, *et al*. Microbiota-induced TNF-like ligand 1A drives group 3 innate lymphoid cell-mediated barrier protection and intestinal T cell activation during colitis. Immunity. 2018;49(6):1077–1089. doi:10.1016/j.immuni.2018.10.014. 30552020 PMC6301104

[14] Brestoff JR, Kim BS, Saenz SA, Stine RR, Monticelli LA, Sonnenberg GF, Thome JJ, Farber DL, Lutfy K, Seale P, *et al*. Group 2 innate lymphoid cells promote beiging of white adipose tissue and limit obesity. Nature. 2015;519(7542):242–246. doi:10.1038/nature14115. 25533952 PMC4447235

[15] Lee B-C, Kim M-S, Pae M, Yamamoto Y, Eberlé D, Shimada T, Kamei N, Park H-S, Sasorith S, Woo J, *et al*. Adipose natural killer cells regulate adipose tissue macrophages to promote insulin resistance in obesity. Cell Metab. 2016;23(4):685–698. doi:10.1016/j.cmet.2016.03.002. 27050305 PMC4833527

[16] O’Sullivan TE, Rapp M, Fan X, Weizman O-E, Bhardwaj P, Adams NM, Walzer T, Dannenberg AJ, Sun JC. Adipose-resident group 1 innate lymphoid cells promote obesity-associated insulin resistance. Immunity. 2016;45(2):428–441. doi:10.1016/j.immuni.2016.06.016. 27496734 PMC5004886

[17] Wang H, Shen L, Sun X, Liu F, Feng W, Jiang C, Chu X, Ye X, Jiang C, Wang Y, *et al*. Adipose group 1 innate lymphoid cells promote adipose tissue fibrosis and diabetes in obesity. Nat Commun. 2019;10(1):3254. doi:10.1038/s41467-019-11270-1. 31332184 PMC6646407

[18] Sasaki T, Moro K, Kubota T, Kubota N, Kato T, Ohno H, Nakae S, Saito H, Koyasu S. Innate lymphoid cells in the induction of obesity. Cell Rep. 2019;28:202-217. 10.1016/j.celrep.2019.06.016 31269440

[19] Boulenouar S, Michelet X, Duquette D, Alvarez D, Hogan AE, Dold C, O’Connor D, Stutte S, Tavakkoli A, Winters D, *et al*. Adipose type one innate lymphoid cells regulate macrophage homeostasis through targeted cytotoxicity. Immunity. 2017;46:273–286. doi:10.1016/j.immuni.2017.01.008. 28228283

[20] Okamura T, Hashimoto Y, Majima S, Senmaru T, Ushigome E, Nakanishi N, Asano M, Yamazaki M, Takakuwa H, Hamaguchi M, *et al*. Trans fatty acid intake induces intestinal inflammation and impaired glucose tolerance. Front Immunol 2021;12.https://www.frontiersin.org/articles/10.3389/fimmu.2021.669672 (accessed 19 Aug 2022). 10.3389/fimmu.2021.669672 PMC8117213 33995404

[21] Li J, Doty AL, Iqbal A, Glover SC. The differential frequency of Lineage(-)CRTH2(-)CD45(+)NKp44(-)CD117(-)CD127(+)ILC subset in the inflamed terminal ileum of patients with Crohn’s disease. Cell Immunol. 2016;304–305:63–68. doi:10.1016/j.cellimm.2016.05.001. 27215784

[22] Forkel M, van Tol S, Höög C, Michaëlsson J, Almer S, Mjósberg J. Distinct alterations in the composition of mucosal innate lymphoid cells in newly diagnosed and established crohn’s disease and ulcerative colitis. J Crohns Colitis. 2019;13:67–78. doi:10.1093/ecco-jcc/jjy119. 30496425

[23] Wensveen FM, Jelenčić V, Valentić S, Šestan M, Wensveen TT, Theurich S, Glasner A, Mendrila D, Štimac D, Wunderlich FT, *et al*. NK cells link obesity-induced adipose stress to inflammation and insulin resistance. Nat Immunol. 2015;16:376–385. doi:10.1038/ni.3120. 25729921

[24] Taouis M, Benomar Y. Is resistin the master link between inflammation and inflammation-related chronic diseases? Mol Cell Endocrinol. 2021;533:111341. doi:10.1016/j.mce.2021.111341. 34082045

[25] Obradovic M, Sudar-Milovanovic E, Soskic S, Essack M, Arya S, Stewart AJ, Gojobori T, Isenovic ER. Leptin and obesity: role and clinical implication. Front Endocrinol (Lausanne). 2021;12:585887. doi:10.3389/fendo.2021.585887. 34084149 PMC8167040

[26] Everard A, Lazarevic V, Gaïa N, Johansson M, Ståhlman M, Backhed F, Delzenne NM, Schrenzel J, François P, Cani PD, *et al*. Microbiome of prebiotic-treated mice reveals novel targets involved in host response during obesity. ISME J. 2014;8(10):2116–2130. doi:10.1038/ismej.2014.45. 24694712 PMC4163056

[27] Lin Y-H, Luck H, Khan S, Schneeberger PHH, Tsai S, Clemente-Casares X, Lei H, Leu Y-L, Chan YT, Chen H-Y, *et al*. Aryl hydrocarbon receptor agonist indigo protects against obesity-related insulin resistance through modulation of intestinal and metabolic tissue immunity. Int J Obes (Lond). 2019;43(12):2407–2421. doi:10.1038/s41366-019-0340-1. 30944419 PMC6892742

[28] Lebrun LJ, Lenaerts K, Kiers D, Pais de Barros J-P, Le Guern N, Plesnik J, Thomas C, Bourgeois T, Dejong CHC, Kox M, *et al*. Enteroendocrine L cells sense LPS after gut barrier injury to enhance GLP-1 secretion. Cell Rep. 2017;21(5):1160–1168. doi:10.1016/j.celrep.2017.10.008. 29091756

[29] Cani PD, Possemiers S, Van de Wiele T, Guiot Y, Everard A, Rottier O, Geurts L, Naslain D, Neyrinck A, Lambert DM, *et al*. Changes in gut microbiota control inflammation in obese mice through a mechanism involving GLP-2-driven improvement of gut permeability. Gut. 2009;58(8):1091–1103. doi:10.1136/gut.2008.165886. 19240062 PMC2702831

[30] Deacon CF. Physiology and pharmacology of DPP-4 in glucose homeostasis and the treatment of type 2 diabetes. Front Endocrinol (Lausanne). 2019:10. doi:10.3389/fendo.2019.00080. PMC6384237 30828317

[31] Romacho T, Sell H, Indrakusuma I, Roehrborn D, Castañeda TR, Jelenik T, Markgraf D, Hartwig S, Weiss J, Al-Hasani H, *et al*. DPP4 deletion in adipose tissue improves hepatic insulin sensitivity in diet-induced obesity. American J Physio Endocrinol Meta. 2020;318(5):E590–9. doi:10.1152/ajpendo.00323.2019. 31891536

[32] Natividad JM, Lamas B, Pham HP, Michel M-L, Rainteau D, Bridonneau C, da Costa G, van Hylckama Vlieg J, Sovran B, Chamignon C, *et al*. Bilophila wadsworthia aggravates high fat diet induced metabolic dysfunctions in mice. Nat Commun. 2018;9(1):2802. doi:10.1038/s41467-018-05249-7. 30022049 PMC6052103

[33] Plovier H, Everard A, Druart C, Depommier C, Van Hul M, Geurts L, Chilloux J, Ottman N, Duparc T, Lichtenstein L, *et al*. A purified membrane protein from Akkermansia muciniphila or the pasteurized bacterium improves metabolism in obese and diabetic mice. Nat Med. 2017;23(1):107–113. doi:10.1038/nm.4236. 27892954

[34] Molinaro A, Bel Lassen P, Henricsson M, Wu H, Adriouch S, Belda E, Chakaroun R, Nielsen T, Bergh PO, Rouault C, *et al*. Imidazole propionate is increased in diabetes and associated with dietary patterns and altered microbial ecology. Nat Commun. 2020;11. doi:10.1038/s41467-020-19589-w. PMC7676231 33208748

[35] Lee L-C, Hou Y-C, Hsieh -Y-Y, Chen Y-H, Shen Y-C, Lee I-J, Monica Shih M-C, Hou W-C, Liu H-K. Dietary supplementation of rutin and rutin-rich buckwheat elevates endogenous glucagon-like peptide 1 levels to facilitate glycemic control in type 2 diabetic mice. J Funct Foods. 2021;85:104653. doi:10.1016/j.jff.2021.104653.

[36] Le TT, Ziemba A, Urasaki Y, Hayes E, Brotman S, Pizzorno G. Disruption of uridine homeostasis links liver pyrimidine metabolism to lipid accumulation. J Lipid Res. 2013;54(4):1044–1057. doi:10.1194/jlr.M034249. 23355744 PMC3605981

[37] Chen T, Qin Y, Chen M, Zhang Y, Wang X, Dong T, Chen G, Sun X, Lu T, White RA, *et al*. Gestational diabetes mellitus is associated with the neonatal gut microbiota and metabolome. BMC Med. 2021;19(1):120. doi:10.1186/s12916-021-01991-w. 34039350 PMC8157751

[38] Liu Y, Xie C, Zhai Z, Deng Z-Y, De Jonge HR, Wu X, Ruan Z. Uridine attenuates obesity, ameliorates hepatic lipid accumulation and modifies the gut microbiota composition in mice fed with a high-fat diet. Food Funct. 2021;12:1829–1840. doi:10.1039/D0FO02533J. 33527946

[39] Steculorum SM, Paeger L, Bremser S, Evers N, Hinze Y, Idzko M, Kloppenburg P, Brüning J. Hypothalamic UDP increases in obesity and promotes feeding via P2Y6-dependent activation of AgRP neurons. Cell. 2015;162(6):1404–1417. doi:10.1016/j.cell.2015.08.032. 26359991

[40] Matsumoto A, Kanai T, Mikami Y, Chu P, Nakamoto N, Ebinuma H, Saito H, Sato T, Yagita H, Hibi T, *et al*. IL-22-producing RORγt-dependent innate lymphoid cells play a novel protective role in murine acute hepatitis. PLOS ONE. 2013;8(4):e62853. doi:10.1371/journal.pone.0062853. 23626860 PMC3633830

[41] Olivares M, Rodriguez J, Pötgens SA, Neyrinck AM, Cani PD, Bindels LB, Delzenne NM. The janus face of cereals: wheat-derived prebiotics counteract the detrimental effect of gluten on metabolic homeostasis in mice fed a high-fat/high-sucrose diet. Mol Nutr Food Res. 2019;63:e1900632. doi:10.1002/mnfr.201900632. 31608562 PMC7003472

[42] López-Almela I, Romaní-Pérez M, Bullich-Vilarrubias C, Benítez-Páez A, Gómez Del Pulgar EM, Francés R, Liebisch G, Sanz Y. Bacteroides uniformis combined with fiber amplifies metabolic and immune benefits in obese mice. Gut Microbes. 2021;13:1–20. doi:10.1080/19490976.2020.1865706. PMC8018257 33499721

[43] Liébana-García R, Olivares M, Rodríguez-Ruano SM, Tolosa-Enguís V, Chulia I, Gil-Martínez L, Guillamón E, Baños A, Sanz Y. The allium derivate propyl propane thiosulfinate exerts anti-obesogenic effects in a murine model of diet-induced obesity. Nutrients. 2022;14(3):440. doi:10.3390/nu14030440. 35276798 PMC8839906

