# Supplementary material for: Intestinal group 1 innate lymphoid cells drive macrophage-induced inflammation and endocrine defects in obesity and promote insulinemia
Source: Gut Microbes. 2023 Feb 23;15(1):2181928. doi: 10.1080/19490976.2023.2181928 (PMC9980552; doi:10.1080/19490976.2023.2181928)
Supplement: Supplemental Material [file KGMI_A_2181928_SM1535.zip › Supplementary materialLiebanaGarciaetal2022Reviewed.docx]

**SUPPLEMENTARY MATERIAL**

**DPP4 activity**

The DPP-4 activity was determined by the cleavage of para-nitroanilide (PNA) from the substrate Gly-Pro-PNA (Sigma, Madrid, Spain). Briefly, 20–50 mg of tissue was homogenized in Tris buffer (50 mM, 1% n-octyl-glucoside, pH 8.3). A volume of the supernatant (20 µL) was incubated with Gly-Pro-PNA and enzymatic activity was measured kinetically for 30 minutes at 37°C (380 nm). DPP4 activity was quantified with a standard curve of free PNA. In the adipose tissue and liver samples, the values were normalized by the amount of protein, quantified by the Bradford method (Bio-Rad, Hercules, CA).

**Immune staining**

The epithelium and the lamina propria were isolated, digested, and stained with the following antibodies: ILC1s (CD90^+^ CD127^+^ LIN^-^ Tbet^+^ IFNɤ^+^) in the intraepithelial fraction were labeled with a PerCP-Cy™5.5-conjugated anti-Lineage antibody cocktail (LIN) (BD Biosciences, Franklin Lakes, NJ), phycoerythrin (PE)-Vio615-conjugated anti-Tbe and allophycocyanin (APC)-conjugated anti-IFNɤ (Miltenyi Biotec, Bergisch Gladbach, Germany). Lamina propria ILC2s (CD90^+^ CD127^+^ LIN^-^ GATA3^+^ IL-4^+^) and ILC3s (CD90^+^ CD127^+^ LIN^-^ RORɤt^+^ IL-22^+^) were determined using PerCP-Cy™5.5-conjugated anti-LIN, Brilliant Ultraviolet 395 (BUV395)-conjugated anti-GATA 3 (BD Biosciences), PE-Cy7-conjugated anti-IL-4 (Biolegend, San Diego, CA), VioB515-conjugated anti-RORɤt (Miltenyi Biotec) and PE-conjugated anti-IL-22 (Biolegend) antibodies. Pro-inflammatory (M1: F4/80^+^ CD80^+^ iNOS^+^) and anti-inflammatory (M2: F4/80^+^ CD206^+^ Arg1^+^) macrophages were labeled with FITC-conjugated anti-F4/80^+^, PE-Vio770-conjugated anti-CD80 (Miltenyi Biotec), APC-conjugated anti-iNOS2 (ThermoFisher Scientific), PerCPCy5.5-conjugated anti-CD206 (Biolegend) and PE-conjugated anti-Arg1 (R&D Systems, Minneapolis, MN) antibodies. For all intracellular marker staining, cells were permeabilized and fixed (fixation/permeabilization solution kit, BD Biosciences). Data acquisition and analysis were performed using a BD LSRFortessa flow cytometer operated with FACS Diva software v.7.0 (BD Biosciences). Data were analyzed using FCS express version 5.

**Histological and immunohistochemical analysis**

Adipocyte size was quantified in visceral WAT after staining with hematoxylin/eosin. Bright-field digital images were taken using an Eclipse 90I (E90I) Nikon microscope (Nikon Corp., Tokyo, Japan) equipped with a 5-megapixel cooled digital color camera (DS-5Mc; Nikon Corp.) under a CFI Plan Fluor 4X (MRH00040) Nikon objective with an optical zoom factor of 2×. A combined analysis was carried out using Fiji (ImageJ 1.49q Software, NIH) and NIS-elements BR 3.2 software (Nikon Corp.). The cross-sectional area of each adipocyte was automatically recognized and calculated by the NIS-elements software. Artifacts were manually discarded. Three independent measurements from different sections were performed on each mouse, and 80–350 adipocytes were quantified on each measurement.

Neuroendocrine cells (NECs) in the ileum were detected in paraffin sections (5 μm) immunostained with an antibody to synaptophysin, using the Autostainer link 48 (DAKO, Glostrup, Denmark). The number of immune-positive cells in two non-serial sections was counted by a blind specialist researcher using an E90I Nikon microscope. The mucosa area of the sections was then manually delineated using NIS-elements software and the results were expressed as the average of NEC number per mucosal area (number/mm^2^).

Mucin production in the colon was calculated after staining with Alcian blue using eosin as a contrast dye. Bright-field digital images were taken using an E90I Nikon wide-field microscope, as above. The main individual image properties were as follows: RGB 24 bits, frame size of 2560*1920 pixels, image dimensions of the field of view of 1.08*0.815 mm (0.43 microns/pixel). To analyze the complete colon section, several overlapping images of each intestinal section were stitched automatically with Fiji software (ImageJ 1.49q software). The area positive for Alcian blue and the total mucosal area were selected using a macro with fixed binary segmentation parameters. Both areas were calculated using NIS-elements BR 3.2 software. Mucin production was expressed as the total area of mucin-producer cells relative to the whole mucosa area.

**Metabolic analyses**

Reversed-phase chromatography analysis was performed using an Acquity BEH C18 (100 × 2.1 mm, 1.7 μm) column (Waters Corp., Wilmslow, UK) on an Agilent 1290 Infinity UPLC chromatograph (Agilent Technologies, Santa Clara, CA). Full scan MS data from 100 to 1700 *m/z* was collected on an iFunnel quadrupole time-of-flight (TOF) Agilent 6550 spectrometer (Agilent Technologies). Electrospray ionization was used for sample volatilization. ESI+ and ESI- analysis were carried out in independent batches. Peak table generation was carried out for each batch separately using XCMS in RStudio (RStudio Inc., Boston, MA). Metabolite annotation was performed using an iterative data-dependent acquisition (DDA) method previously developed^1^, and matching *m/z* values, isotopic profiles, and MS/MS data against the Human Metabolome Database (www.hmdb.ca), METLIN and LipidBlast (LipiDex) databases. Data acquisition and manual integration were carried out employing the MassHunter Workstation (version B.07.00) from Agilent. Raw data were converted into mzXML format using ProteoWizard (http://proteowizard.sourceforge.net/). Peak detection, integration, deconvolution, alignment, and pseudospectral identification were carried out using XCMS and CAMERA in R v.4.2.

For reversed-phase chromatography, autosampler and column temperatures were set to 4°C and 55°C, respectively, and the injection volume used was 4 μl. Gradient elution was performed at a flow rate of 400 μl/min as follows: initial conditions of 98% of mobile phase A (H_2_O, 0.1% v/v HCOOH) followed by a linear gradient, 2–20%, of mobile phase B (CH_3_CN, 0.1% v/v HCOOH) in 3.5 min and 20–95% B in 4 min. The 95% elution of phase B was held for 1 min and then a gradient of 0.1 min was used to return to the initial conditions, which were held for an additional 2.4 min. Standard needle washes were carried out between injections using the mobile phase to reduce autosampler carryover between injections. The following electrospray ionization parameters were selected: gas temperature, 200°C; drying gas, 14 l/min; nebulizer, 37 psig; sheath gas temperature, 350°C; sheath gas flow, 11 l/min. Automatic MS spectra calibration during the analysis was carried out by introducing a reference standard into the source via a reference sprayer valve and using the following ions (*m/z*) as references: 149.02332, 121.050873 and 922.009798 (positive mode), and 119.036 and 980.0163 (negative mode). Data obtained during initial column conditioning were excluded from data analysis. For peak detection, the centWave algorithm was used with the following parameters: mass accuracy, 15 ppm; peak width, (5,20); snthresh, 6; prefilter, (3,1000). Intensity weighted *m/z* values of each feature were calculated using the mean function. Peak limits used for integration were found through descent on the Mexican hat-filtered data. Grouping before and after retention time correction was carried out using the density method. Missing data points were filled by reintegrating the raw data files in the regions of the missing peaks using the fill Peaks method. Accuracy of the peak integration and alignment were assessed by comparing peak area values obtained by manual and automated (i.e., XCMS) integration results for deuterated injection standards and known metabolites, obtaining linear correlation coefficients > 0.99. A set of 11 QC replicates were injected at the beginning of each batch to ensure system conditioning, and a QC was analyzed every 6 samples. Three consecutive blanks were analyzed at the end of each batch for background elimination. QC replicates were used for the correction of within-batch effects using the quality control samples and support vector regression correction algorithm^2,3^ and a radial basis function kernel. Selection of the tolerance threshold (ε), the penalty term applied to margin slack values (C), and the kernel width (γ) was carried out using a pre-selection of C and optimization of ε and γ using a grid search, leave-one-out cross-validation, and the root mean squared error of cross-validation (RMSECV) as target function. C was selected for each UPLC-MS feature as the median value of the intensities observed in QC replicates. The ε search range was selected based on the expected instrument precision (4–10% of the median value of the intensities observed for the whole set of QC replicates). The γ search interval selected was [1, 10^5^]. UPLC-MS features were removed from further analysis if the relative standard deviation of QCs >20% after batch effect correction. A second data clean-up was carried out after within-batch effect correction using the set of blank samples independently for each batch. For metabolite annotation, an aliquot of the QC was repeatedly analyzed using the auto DDA mode with the following inclusion *m*/*z* precursor ranges, 100–200, 200–400, 400–600, 600–800 and 800–1200, using in all replicates centroid mode at a rate of six spectra/s in the extended dynamic range mode (2 GHz). The collision energy was set to 20 V, MS/MS fragmentation with an automated selection of five precursor ions per cycle and an exclusion window of 0.15 min after two consecutive fragmentations. The MS/MS spectral match was carried out using the geometric mean value of the dot and the reverse dot products. Fragment ions present in the reference spectra included in the same pseudospectrum of an annotated metabolite were annotated as fragments.

The differential concentration analysis of metabolomics was performed with the Wilcoxon Rank Sum test corrected by the Benjamini-Horchberg false discovery rate (BH-FDR) procedure. Principal component analysis (PCA) was performed with the FactoMineR v.2.4 R package, centering and scaling all the detected metabolites based on differences in concentration. Pathway analysis was performed with the mummichog algorithm^4^ with a p-value cut-off of 0.05 and the human KEGG pathway database. Data analysis was carried out in MATLAB 2021a (Mathworks Inc., Natick, MA) using in-house written scripts, and the PLS Toolbox 8.9 (Eigenvector Research Inc., Wenatchee, WA). For the integration analysis, significant features in the comparisons of ILC1-targeted and non-targeted obese mice from the differential abundance analysis were selected in both metabolomic and 16S datasets. Regarding repeated features, non-molecular ions were removed from the list of selected metabolites. Spearman’s correlation between features was calculated with the cor.test function in R and p-values were adjusted by BH-FDR. The correlation matrix was graphically displayed with the corrplot v0.92 R package with hierarchical clustering order using the ward.D agglomeration method.

References:

1 Kuligowski J, Sánchez-Illana Á, Sanjuán-Herráez D, *et al.* Intra-batch effect correction in liquid chromatography-mass spectrometry using quality control samples and support vector regression (QC-SVRC). *The Analyst* 2015;**140**:7810–7. doi:10.1039/c5an01638j

2 Ten-Doménech I, Martínez-Sena T, Moreno-Torres M, *et al.* Comparing targeted vs. untargeted MS2 data-dependent acquisition for peak annotation in LC–MS metabolomics. *Metabolites* 2020;**10**:126.

3 Sánchez-Illana Á, Pérez-Guaita D, Cuesta-García D, *et al.* Model selection for within-batch effect correction in UPLC-MS metabolomics using quality control - Support vector regression. *Anal Chim Acta* 2018;**1026**:62–8. doi:10.1016/j.aca.2018.04.055

4 Li S, Park Y, Duraisingham S, *et al.* Predicting Network Activity from High Throughput Metabolomics. *PLOS Computational Biology* 2013;**9**:e1003123. doi:10.1371/journal.pcbi.1003123

**Supp. Table 3**

Primers used in this study

| **Targeted gene** | **Sequences (5’-3’)** |
| --- | --- |
| *Cd36* | F: GCCAAGCTATTGCGACATGA  R: ATCTCAATGTCCGAGACTTTTCAAC |
| *Cebpb* | F: TCGGGACTTGATGCAATCC  R: AAACATCAACAACCCCGC |
| *Cldn3* | F: TCATCGGCAGCAGCATCATCAC  R: ACGATGGTGATCTTGGCCTTGG |
| *Il17* | F: CTCCAGAAGGCCCTCAGACTAC  R: GGGTCTTCATTGCGGTGG |
| *Il22* | F: GACATAAACAGCAGGTCCAGTT  R: AGAAGGCTGAAGGAGACAGT |
| *Lpl* | F: TGAAAGCCGGAGAGACTCAG  R: AGTGTCAGCCAGACTTCTTCAG |
| *Muc2* | F: CCCAGAAGGGACTGTGTATG  R: TGCAGACACACTGCTCACA |
| *NeuroD1* | F: AGGAATTCGCCCACGCAGAAG  R: CTCCTCTGCATTCATGGCTTCAAG |
| *Ngn3* | F: ACTCAGCAAACAGCGAAGAAG  R: CAGTGCCCAGATGTAGTTGTG |
| *Ocln* | F: ATGTCCGGCCGATGCTCTC  R: TTTGGCTGCTCTTGGGTCTGTAT |
| *Pla2ga* | F: AAGGATCCCCCAAGGATGCCAC  R: CAGCCGTTTCTGACAGTTCTGG |
| *Reg3g* | F: TTCCTGTCCTCCATGATCAAA  R: CATCCACCTCTGTTGGGTTC |
| *Tcf 4* | F: ATGGCAAACAGAGGAACTGG  R: GCCTGCTGAGAGTGAAGGAG |
| *Zo1* | F: TTTTTGACAGGGGGAGTGG  R: TGCTGCAGAGGTCAAAGTTCAAG |

**
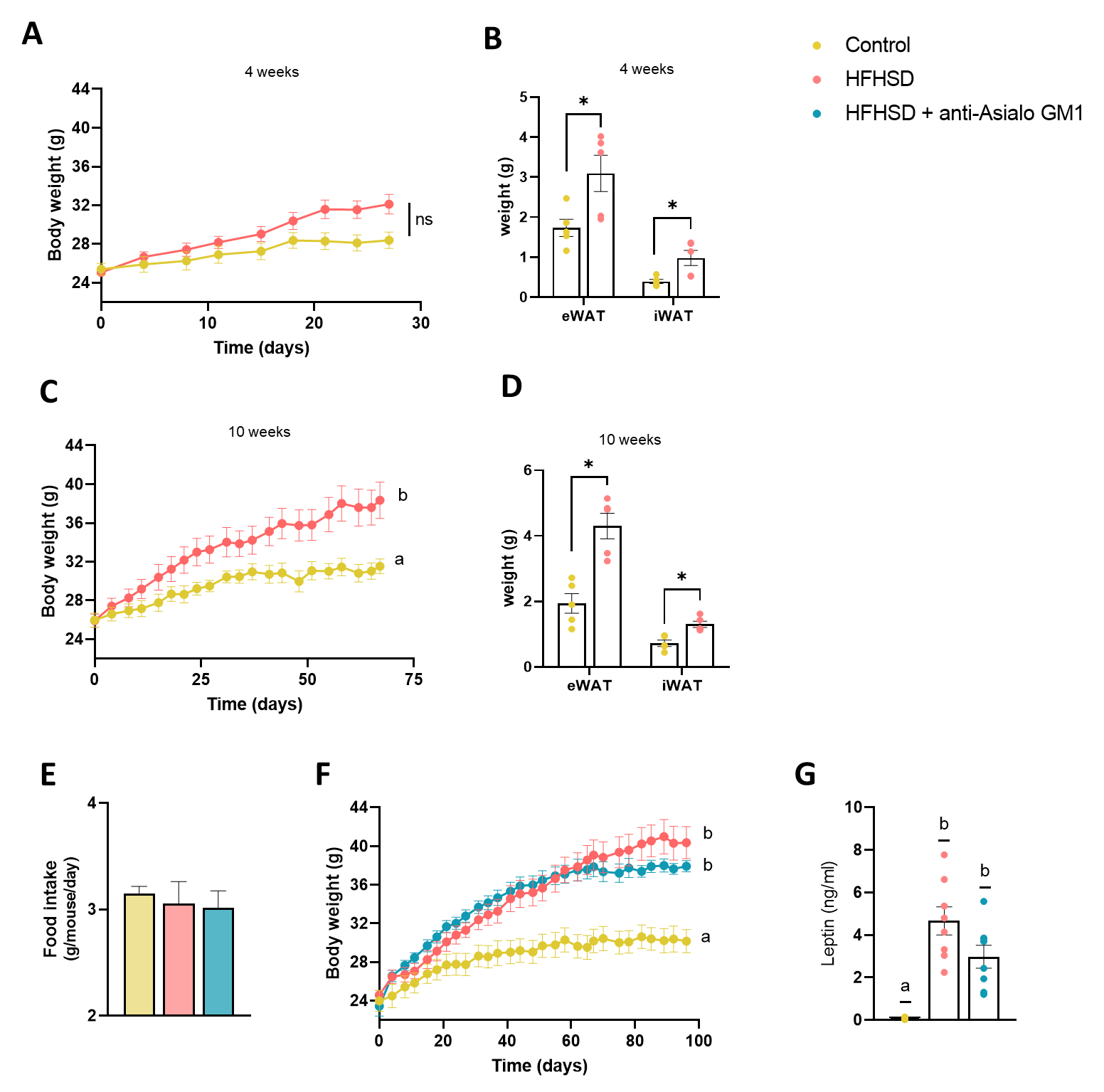
**

**Supp. Fig 1.** Changes in body weight and adiposity after exposure to an obesogenic diet. **(A)** Body weight evolution and **(B)** percentage of epididymal (eWAT) and inguinal (iWAT) white adipose tissue after 4 weeks of HFHSD (n=5), **(C)** body weight evolution and, **(D)** percentage of eWAT and iWAT after 10 weeks of HFHSD (n=5), **(E)** food intake from week 10 to week 14 in HFHSD-fed mice and **(F)** body weight evolution after 14 weeks of HFHSD (n=8) **(G)** Plasma leptin levels. Bars represent the mean ± SEM and biological replicates are shown as individual dots. Changes in body weight were analyzed by two-way ANOVA followed by Tukey’s post hoc test. Changes in adiposity were assessed by using a t-test. Food intake and leptin levels were analyzed by one-way ANOVA. Different superscript letters or the symbol “*” show statistical differences when p < 0.05.

**
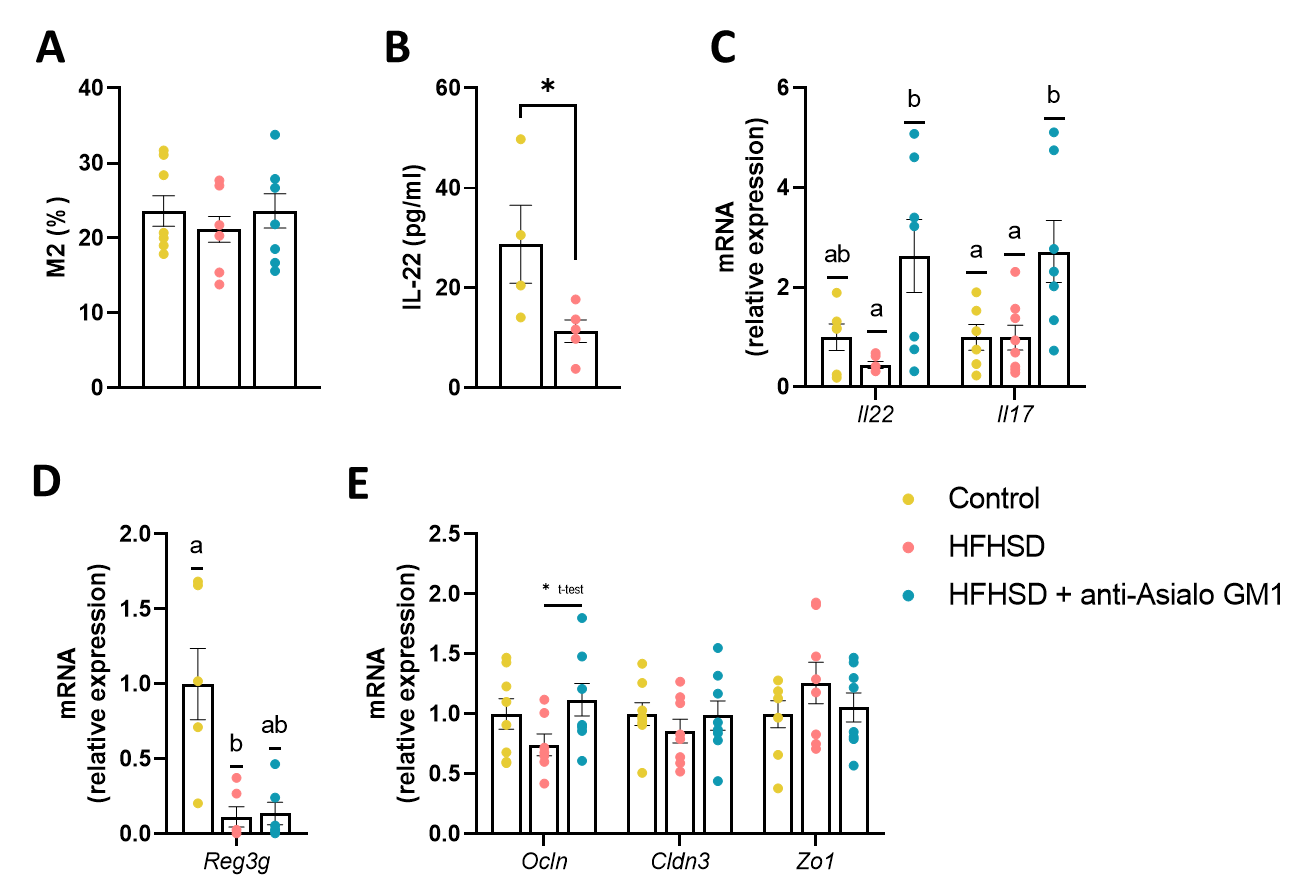
**

**Supp. Fig 2.** Changes in immune mediators. **(A)** Anti-inflammatory M2 macrophages (percentage of F4/80^+^ cells of total lamina propria cells) in mice fed a HFHSD for 14 weeks (n=8) **(B)** Plasma levels of IL-22 after 10 weeks of an HFHSD (n=5) **(C)** Gene expression of IL-22 and IL-17 in the ileum (n=6-8), **(D)** Gene expression of regenerating islet-derived 3-gamma (*Reg3g*) in colon (n=6-8) and **(E)** Gene expression of tight junction proteins in the colon (n=7-8)**.** Bars represent the mean ± SEM and biological replicates are shown as individual dots. The M2 (A) and *Reg3g* expression changes (D) were analyzed with the Kruskal–Wallis test, changes in IL-22 by an unpaired t-test and ileal (C) and colonic (E) relative expression changes analyzed by One-Way ANOVA. Different superscript letters or the symbol “*” shows significant differences at p < 0.05.


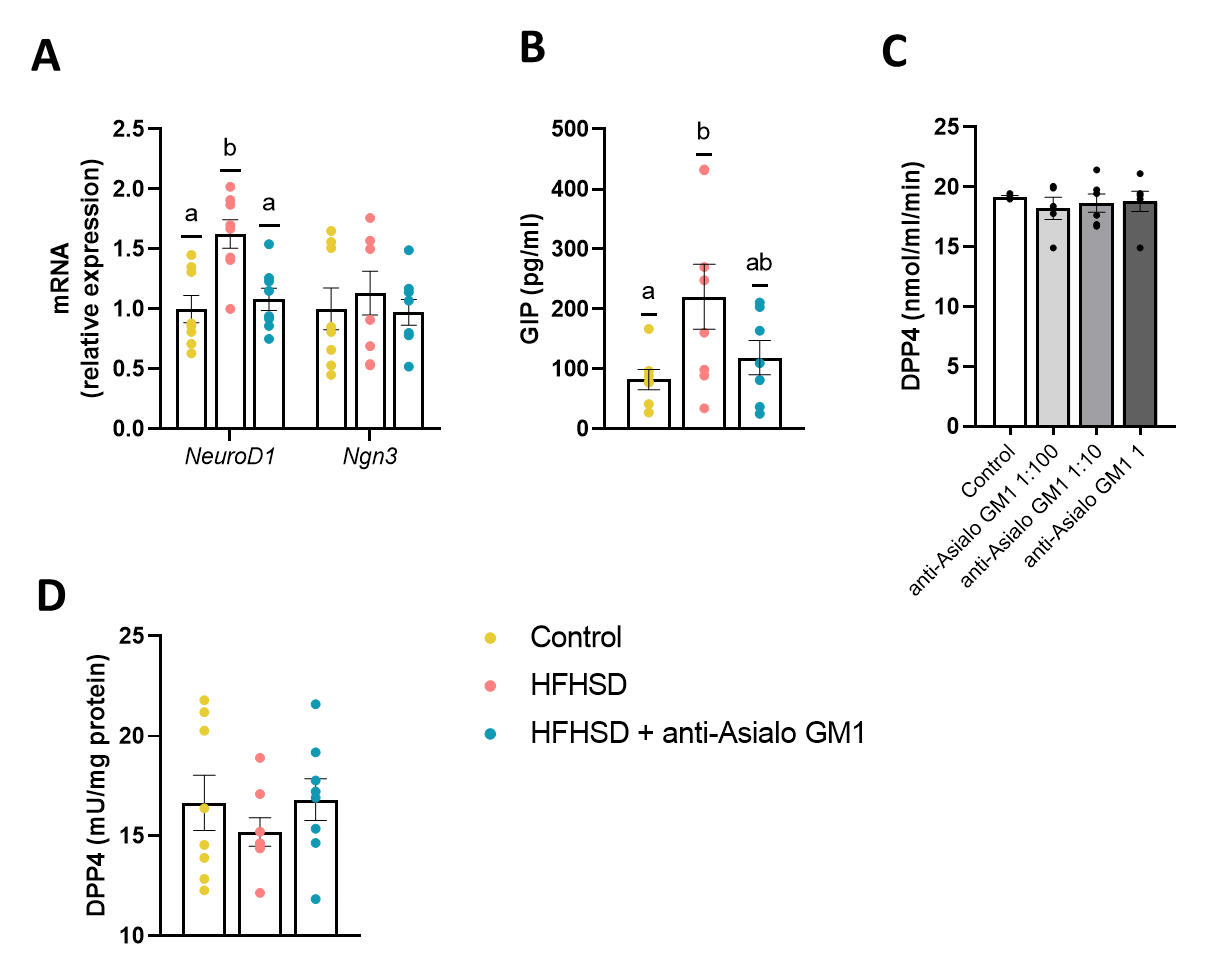


**Supp. Fig 3.** Changes in endocrine markers in mice. **(A)** Gene expression of the indicated factors of enteroendocrine cell differentiation in the ileum (n=8). **(B)** Plasma GIP levels (n=7–8). **(C)** DPP4 activity in a pool of plasma with or without different dilutions of anti-Asialo GM1 (n=4–6). **(D)** DPP4 activity in the liver (n=8). Bars represent the mean ± SEM and biological replicates are shown as individual dots. Data were analyzed by one-way ANOVA. Data with different superscript letters are significantly different at p < 0.05.


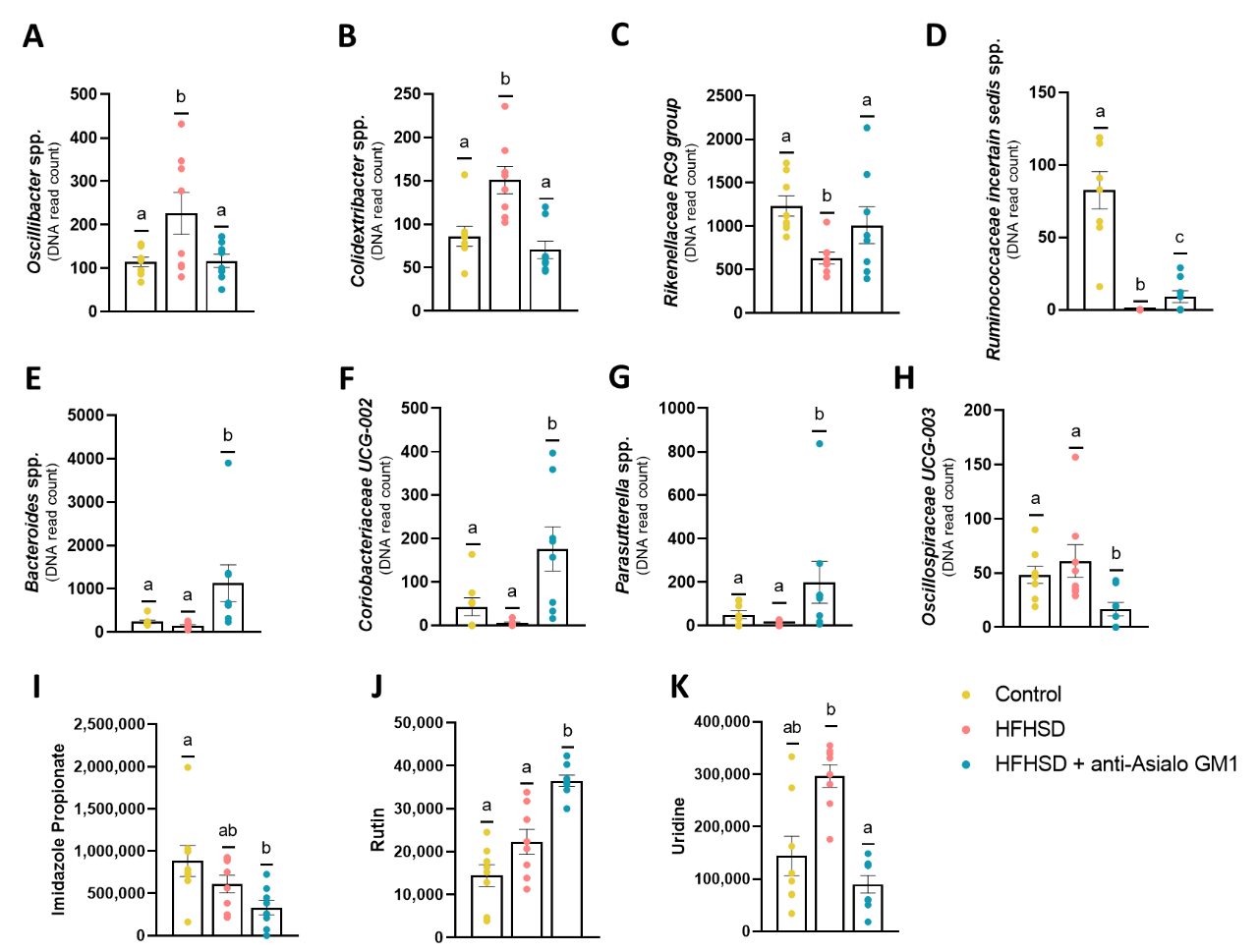


**Supp. Fig 4**. Bacterial abundance and metabolites in the cecal content. **(A)** *Oscillibacter* spp., **(B)** *Colidextribacter* spp., **(C)** *Rikenellaceae* RC9 group, **(D)** *Ruminococcaceae incertain sedis* spp., **(E)** *Bacteroides* spp., **(F)** *Coriobacteriaceae* UCG-002, **(G)** *Parasutterella* spp*.,* **(H)** *Oscillospiraceae* UCG-03*,* **(I)** imidazole propionate (arbitrary units), **(J)** rutin (arbitrary units) and **(K)** uridine (arbitrary units). n=8 in all cases. Bars represent the mean ± SEM and biological replicates are shown as individual dots. Differential metabolite abundance between groups were compared with a Wilcoxon rank sum test and the p-value was corrected with the Benjamini-Hochberg procedure. Data with different superscript letters are significantly different at p < 0.05.
